# EIT Imaging of Intracranial Hemorrhage in Rabbit Models Is Influenced by the Intactness of Cranium

**DOI:** 10.1155/2018/1321862

**Published:** 2018-11-19

**Authors:** Meng Dai, Xue-Chao Liu, Hao-Ting Li, Can-Hua Xu, Bin Yang, Hang Wang, Xue-Tao Shi, Xiu-Zhen Dong, Feng Fu

**Affiliations:** ^1^School of Biomedical Engineering, Air Force Military Medical University, Xi'an, China; ^2^School of Aerospace Medicine, Air Force Military Medical University, Xi'an, China

## Abstract

Electrical impedance tomography (EIT) has been shown to be a promising, bedside imaging method to monitor the progression of intracranial hemorrhage (ICH). However, the observed impedance changes within brain related to ICH differed among groups, and we hypothesized that the cranium intactness (open or closed) may be the one of potential reasons leading to the difference. Therefore, the aim of this study was to investigate this effect of open or closed cranium on impedance changes within brain in the rabbit ICH model. In this study, we first established the ICH model in 12 rabbits with the open cranium and in 12 rabbits with the closed cranium. Simultaneously, EIT measurements on the rabbits' heads were performed to record the impedance changes caused by injecting the autologous nonheparinized blood into cerebral parenchyma. Finally, the regional impedance changes on EIT images and the whole impedance changes were analyzed. It was surprisingly found that when the cranium was open, the impedance of the area where the blood was injected, as well as the whole brain impedance, decreased with the amount of blood being injected; when the cranium was closed, while the impedance of the area where blood was not injected continued to increase, the impedance of the area where blood was injected decreased within 20s of the blood being injected and then remained almost unchanged, and the whole brain impedance had a small fall and then notably increased. The results have validated that the cranium completeness (open or closed) has influences on impedance changes within brain when using EIT to monitor ICH. In future study on application of EIT to monitor ICH, the cranium completeness should be taken into account for establishing an ICH model and analyzing the corresponding EIT results.

## 1. Introduction

Intracranial hemorrhage (ICH) is a severe condition where blood suddenly leaks out of a small artery in the brain and pushes into the brain tissue. ICH is commonly caused by long-standing high blood pressure and by arteriovenous malformations, aneurysms, tumors, or traumatic brain injury [1, 2]. It is a life-threatening medical emergency and the deadliest and most disabling type of stroke [3]. According to meta-analysis, less than a half of the patients with ICH survive for one year and 75% of survivors cannot live independently after 12 months [4, 5]. Clinical investigations on ICH have showed early intervention (such as cranial surgery) was associated with more favorable outcome, including reducing mortality and improving neurological outcomes. Therefore, early diagnosis and intervention by clinical staff are of the utmost importance for significantly improving the prognosis of ICH patients.

However, the prompt detection and diagnosis of ICH still remain challenging. Although the current clinical imaging tools, such as computed tomography and magnetic resonance imaging, can accurately diagnose ICH (including identifying the site of bleeding and the amount of blood released) by recording the intracranial anatomical image at a specific point of time [6, 7], these examinations are performed only once on the patients with suspected ICH and often show hematomas many hours after bleeding onset. Also, it is impractical because of the radioactivity of CT scan and the fact that it is a huge waste of medical resources to use CT and MRI scans for the purpose of continuous ICH monitoring [8]. Unfortunately, in the clinical practices, the risk of a hematoma expanding after primary ICH remains approximately at 30% [9]; postoperative secondary bleeding is also common [10, 11]. In such cases, monitoring the development of ICH in real time is desirable. Therefore, a convenient and noninvasive medical equipment capable of immediately detecting and continuously monitoring ICH is urgently needed.

Electrical impedance tomography (EIT) is a real-time, portable, noninvasive, functional imaging technique, which aims to reconstruct the electrical impedance distribution (or the change in electrical impedance) inside the human body by safely injecting a set of currents into the body via surface electrodes and measuring the resulting voltages from different types of tissues [12]. Because there are significant differences in impedance between blood (hematoma) and normal brain tissue [13, 14], EIT can potentially be used to detect and monitor ICH at bedside.

Up to now, several studies had investigated the feasibility of using EIT to monitor ICH in animal models and demonstrated that EIT is able to reflect the impedance changes owing to ICH [15–21]. Interestingly, the results of these studies differ from each other. On the one hand, Xu et al. [15] and Manwaring et al. [16] observed a brain impedance fall in the open cranium pig models when autologous blood was injected into cerebral parenchyma. This finding was also observed by Dowrick et al. [17] who injected autologous blood into rat brain with the closed cranium. On the other hand, Sadleir et al. [18] and Tang et al. [19] employed EIT to monitor ICH in the open/closed cranium pig model of intraventricular hemorrhage and found that brain impedance increased with the amount of blood injected. Dai et al. [20] also reported an increase in brain impedance when autologous blood was injected into subarachnoid space in the closed cranium pig models. Chen et al. [21] recorded the impedance increase of the closed cranium rabbit brain using EIT wherein ICH was induced by injecting collagenase into the white matter. This phenomenon was further investigated by Yang et al. [22] who measured the impedance changes of the closed cranium rabbit brain using impedance analyzer when the autologous blood was also injected into cerebral parenchyma and found that the brain impedance went down firstly and then went up. By comparing the experimental conditions of those studies, we find that the one of the underlying factors contributing to differences between results might be characteristics of the cranium (open or closed) in the ICH model. In clinical setting, the hospitalized ICH patients generally receive the treatment of surgery (open cranium) or conservative medication (closed cranium) according to severity of bleeding. Nevertheless, they are all at risk of secondary bleeding. Therefore, before applying EIT to monitor ICH progression at bedside, it is necessary to investigate the effect of open or closed cranium on impedance changes within brain in the animal ICH model.

In this study, we first established the ICH models with open or closed cranium by injecting the blood into the white matter in rabbits. Second, changes in brain impedance were continuously monitored with EIT throughout the whole process of injecting blood. Finally, the differences in brain impedance change between these results and those of previous studies are analyzed and discussed.

## 2. Materials and Methods

### 2.1. Ethical Statement

All animal experiments in this study were approved by the Ethics Committee for Animal Research of the Air Force Medical University, Xi'an, Shaanxi, People's Republic of China.

### 2.2. Animal Preparation

24 New Zealand white rabbits were divided into two groups: the open cranium ICH group and the closed cranium ICH group. All animals were obtained from the Animal Experimental Center of the Air Force Military Medical University. They were two months old and weighed 2.3 ± 0.4 kg. The rabbits were allowed to drink water until 4 h before the experiment and were fasted for 2 h before the experiment. Abdominal anesthesia was performed with 1.5% pentobarbital sodium (2 mL · kg^−1^). Once a level of sedation was achieved, 3% pentobarbital sodium (0.5 mL · kg^−1^) was injected into the ear rim vein to obtain deep anesthesia. During surgery, 1.5% pentobarbital sodium was injected into the abdominal cavity at a rate of 1 mL · kg^−1^ · h^−1^ to maintain anesthesia. Animal body temperature was measured with a rectal thermistor probe, and a warm water blanket was used to maintain a body temperature of 39.5 ± 0.5°C. Each animal was immobilized onto a stereotactic frame in a prone position using eye- and ear-fixing bars.

### 2.3. Surgery

**Figure 1:**
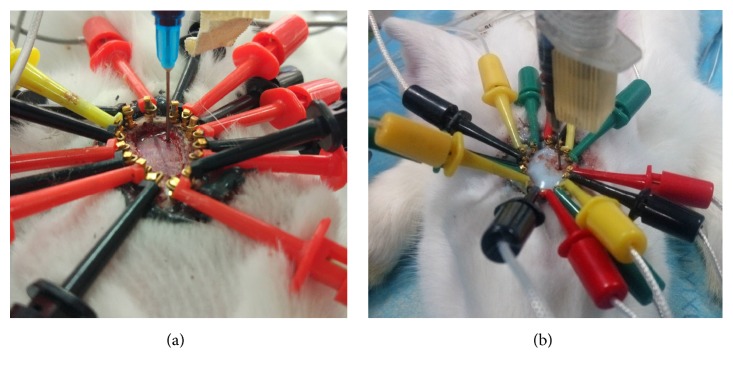
Localized intracranial hemorrhage (ICH) models. (a) Open cranium. (b) Closed cranium.

Before establishing the ICH model, the hair on the rabbit's head was shaved to make approximately 15 cm^2^ of the scalp bald. Then, the bald scalp and underlying periosteum were removed with a scalpel. Hemostasis by electrocoagulation and cleaning up the wound achieved a near-elliptical window, which was approximately 3.6 cm long in the sagittal direction and approximately 2.5 cm long in the coronal direction (see Figure 1). To minimize water loss from the cranium and wound, an even layer of bone wax was smeared on the exposed cranium; an even layer of medical glue was smeared on the exposed cranium and wound. In this study, the ICH models were established by injecting the autologous nonheparinized blood to imitate the clinical circumstance.

#### 2.3.1. Open Cranium ICH Model

12 rabbits were included in this group. In the open cranium ICH model, a dental bur (10 mm in diameter) was used to drill the outer, middle, and inner laminae of the cranium to expose the dura mater, resulting in an elliptical window approximately 3 cm long in the sagittal direction and approximately 2 cm long in the coronal direction. Hemostasis was achieved by electrocoagulation and the wound was cleaned up (see Figure 1(a)); 2 mL of autologous blood was drawn from the heart, and 0.5 mL of blood was aspirated into a 1-mL syringe. The syringe was then fixed onto the stereotactic frame and the needle was inserted into the coronal suture into the cranial cavity 5 mm left to the sagittal suture and 5 mm posterior, at a depth of 8 mm. The injection of blood started 1 min after the needle was inserted; blood was then injected slowly over 150 s.

#### 2.3.2. Closed Cranium ICH Model

There were 12 rabbits included in this group. In the closed cranium ICH model, each animal was immobilized onto a stereotactic frame, and the cranium was drilled using a dental bur 5 mm left to the sagittal suture and 5 mm posterior to the coronal suture to make a hole as deep as the dura mater and 1 mm wide (see Figure 1(b)); 1 mL of autologous blood was drawn from the heart, and 0.5 mL of blood was aspirated into a 1-mL syringe. The syringe was then fixed onto the stereotactic frame and the needle was inserted through the hole into the cranial cavity at a depth of 11 mm, a depth that allowed blood to be injected into the brain parenchyma; afterwards, in order to ensure the closure of cranium, the connection between the needle and the hole was coated again with bone wax. The injection of blood started 1 min after the needle was inserted; blood was then injected slowly over 150 s.

### 2.4. EIT Data Acquisition and Imaging

#### 2.4.1. EIT Imaging: Electrodes, System, and Algorithm

Before establishing the ICH models, 16 electrodes (copper dental nails, 0.93 mm in diameter; Hangzhou Westlake Biomaterial Corporation, Zhejiang, People's Republic of China) were attached to the cranium. Two electrodes were inserted along the sagittal suture, one 2 cm in front and the other 1 cm behind the coronal suture, respectively. The other electrodes were placed at regular intervals along an ellipse that was 3 cm long in the sagittal direction and 2 cm long in the coronal direction. None of the electrodes penetrated the cranium; the depth of the electrodes was approximately 1 mm. Once all the electrodes had been placed, the contact surface of the electrodes and the surface of the cranium were smeared with medical glue (DP100, 3 M Company, Maplewood, MN, USA) to further fix the electrodes (see Figure 1).

EIT data were acquired using a high-precision, multi-frequency EIT system developed in-house [23]. The system operated at 1–190 kHz frequencies and had an output current range of 10 uA–1.25 mA, with a common mode rejection ratio > 75 dB, a relative measurement accuracy of 0.01%, and an acquisition rate of up to 3 frames/s. In this study, the excitation was performed using a sine current at a frequency of 50 kHz and with an amplitude of 500 uA (peak-to-peak); the acquisition rate was set at 1 frame/s. To maintain a good sensitivity and signal-to-noise ratio [24], EIT data were acquired using the opposite excitation/adjacent measurement protocol.

Before blood was injected, reference data were acquired for 1 min. Then, data were acquired in real-time as blood was being injected to monitor changes in electrical impedance in the rabbit brain. EIT images were reconstructed using the damped least squares (DLS) algorithm [25]. The formula of DLS algorithm is shown here:

(1) $$\begin{array}{c} \Delta \rho ={\left(\;S^{T}S+\lambda L\;\right)}^{-1}S^{T}\cdot \Delta V \end{array}$$

where Δ**ρ** is the impedance change between two time points, **S** is the sensitive matrix (the sensitive matrix is calculated using a two-dimensional circular model with even impedance distribution), *λ* is the regularization parameter (here *λ* = 0.1), **L** is the regularization matrix (**L** = diag⁡(**S**^*T*^**S**)), and Δ**V** is the change in boundary voltage.

(2) $$\begin{array}{c} \Delta V=V_{t_{x}}-V_{t_{0}} \end{array}$$

where **V**_*t*_*x*__ is the boundary voltage used to acquire data during blood injection and **V**_*t*_0__ is the reference data (acquired before blood was injected).

#### 2.4.2. EIT Imaging in Saline Solution

EIT imaging was carried out using the cylindrical phantom. The model had a radius of 102.5 mm and 16 electrodes distributed evenly along its periphery. Saline was poured into the model until the liquid was 2 cm above the level of the electrodes. The electrodes were connected to the EIT data acquisition system; then, a copper bar (low impedance) and plastic syringe (high impedance) were inserted into the saline as the perturbed targets. Changes in EIT imaging were observed after the perturbed targets were inserted. EIT imaging in saline solution was carried out before each experiment as a control.

### 2.5. Data Analysis

In order to analyze the change in impedance among various brain regions due to blood being injected, the impedance of the region where blood was injected and the impedance of the region where it was not were calculated, namely, the resistivity variation index (RVI) (RVI_blood_ and RVI_non-blood_, resp.). According to the study by Sadleir et al. [26], the RVI is calculated as follows:

(3) $$\begin{array}{c} RVI=\overset{N}{\underset{i=1}{\sum }}\Delta \rho_{i} \end{array}$$

where Δ**ρ**_*i*_ is the reconstructed impedance value per unit and* N* is the number of units. The region where blood was injected was determined by using the threshold value method. In brief, the maximum reconstructed impedance value, that is, *Z*_max_, was calculated for the whole EIT image; then, all units in the EIT image with a reconstructed impedance value greater than 10% of *Z*_max_ were calculated, and they constituted the hemorrhagic region as the region of interest 1 (ROI 1), that is, ROI_1_ = *Ω*_blood_. All regions other than the hemorrhagic region constituted the region where blood was not injected as the ROI 2, that is, ROI_2_ = *Ω*_non-blood_

(4) Ωblood=Zi>Zmax,  i=1…NΩnon-blood=Ω−blood

Statistically, the difference of RVI_blood_ before and after blood injection was analyzed with independent sample* t*-test using IBM SPSS Statistics for Windows, Version 20.0 (IBM Corporation, Armonk, NY, USA), as well as RVI_non-blood_;* p* < 0.05 was considered significant.

Furthermore, to analyze the ICH-induced change in whole brain resistivity (WBR), the WBR before and after blood was injected was compared. WBR is calculated as follows [17]:

(5) $$\begin{array}{c} WBR=\overset{16}{\underset{i=1}{\sum }}\;\overset{16}{\underset{j=1}{\sum }}\frac{V_{i,j}}{I}\;\left(j\neq 1,4,5,8,9,16\right) \end{array}$$

where *V*_*i*,*j*_ is the measured voltage of the measuring electrodes pair *j*, when the electrode pair *i* was excited, and *I* is the amplitude of the exciting current. Since the contact impedance of the exciting electrodes was unknown, the measured voltage of the exciting electrodes was ignored, that is, *j* ≠ 1,4, 5,8, 9,16. In light of individual differences between animals, the mean WBR within 30 s before blood was injected was calculated for each animal; then, the change in WBR was calculated for the whole period of blood injection using the mean WBR as reference, that is, *WBR*_*reference*_.

(6) $$\begin{array}{c} WBRC_{t}=\frac{WBR_{t}-WBR_{reference}}{WBR_{reference}}\times 100\% \end{array}$$

where *WBR*_*t*_ is the WBR at time point *t* and *WBRC*_*t*_ is the change percentage in WBR at time point *t* in relation to the value before blood was injected.

### 2.6. ICH Model Validation

To validate the ICH models, the needle was withdrawn 5 min after the injection of blood was completed. Each animal was then sacrificed by administering an anesthesia overdose. Then, the cranium was opened to obtain the brain, which was cut open along the needle line. The distribution of the injected blood was observed. Then, a sliver of hemorrhagic tissue (1 cm in diameter) was immersed into 10% formaldehyde. Twenty-four hours later, the tissue specimen was sliced into 3-mm thick sections. The sections were stained with hematoxylin & eosin and analyzed by a pathologist.

## 3. Results

### 3.1. Anatomical Structure and Pathological Results of the ICH Model

**Figure 2:**
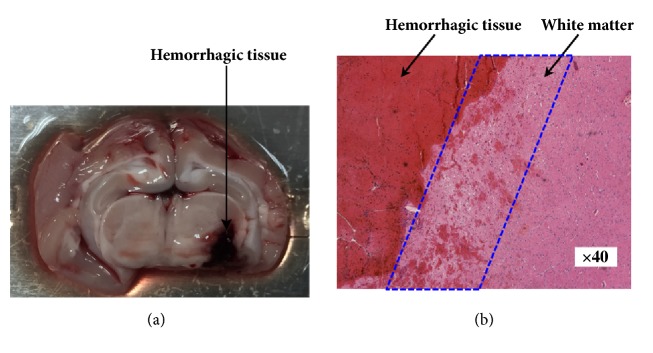
The ICH model. (a) Anatomical structure of the ICH model. (b) Pathological results of the ICH model.

During all experiments, the body temperature and respiration of the animals were kept stable. The ICH model established in the experiments is shown in Figure 2(a); the volume of hemorrhagic tissue (blood clot) was approximately 260 ± 35 mm^3^.  Figure 2(b) shows the hemorrhagic tissue and surrounding normal white matter. The boundary between hemorrhagic tissue and white matter is clear and a few blood cells have penetrated the white matter. These results demonstrated that the ICH model was successfully established in our experiments.

### 3.2. EIT Imaging in Saline Solution

**Figure 3:**
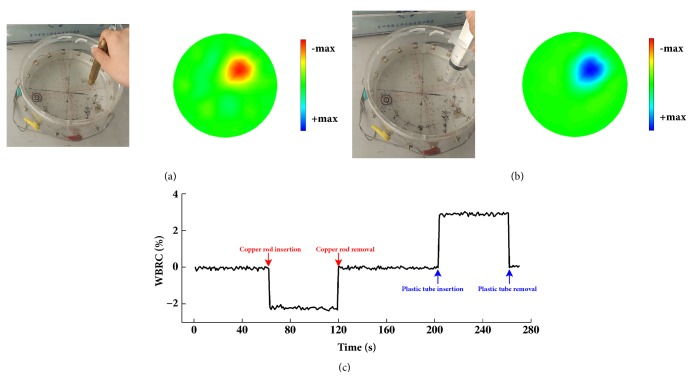
EIT imaging in saline solution. (a) When the copper rod is inserted. (b) When the plastic syringe is inserted. (c) Change in WBR when the copper rod (red) and plastic syringe (blue) were inserted into the saline solution.

Because the impedance of the copper rod is much lower than that of the saline solution, impedance decreased when the copper rod was inserted into the solution and increased when it was removed (see Figure 3(a)); because the impedance of the plastic syringe is much higher than that of the saline solution, impedance increased when the plastic syringe was inserted into the solution and decreased when it was removed (see Figure 3(b)).  Figure 3(c) shows that raw impedance decreased and increased when the copper rod and plastic syringe, respectively, were inserted into the saline solution. These results validated the accuracy and reliability of the EIT system.

### 3.3. EIT Imaging of the Blood Injection Procedure in Rabbits with Open Cranium

**Figure 4:**
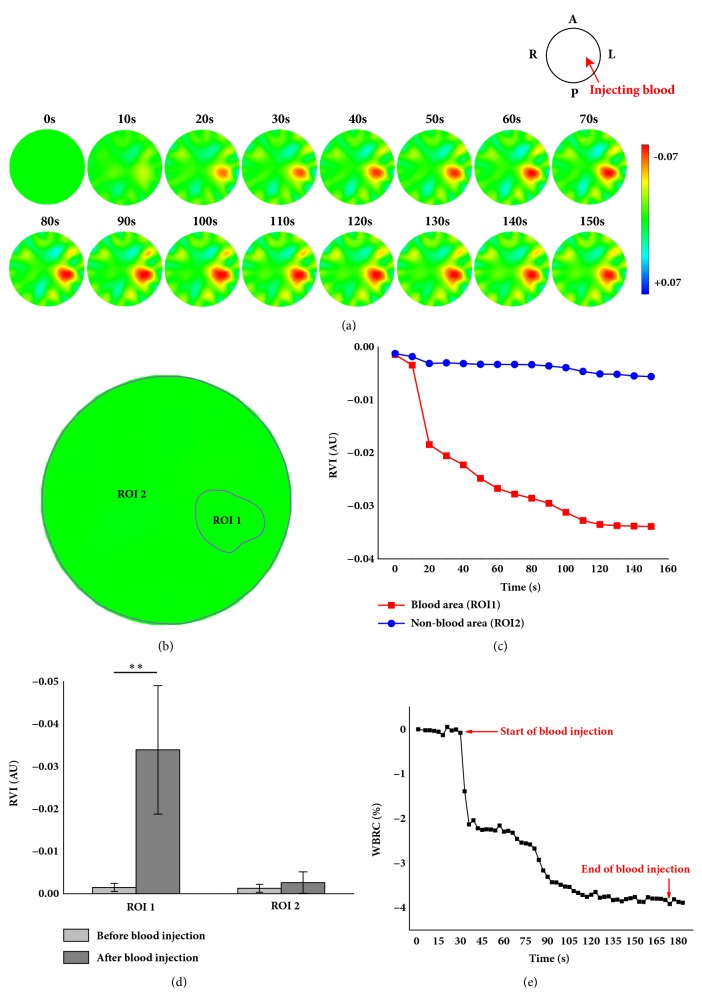
EIT imaging of the ICH procedure in rabbits with the open cranium. (a) The reconstructed EIT images of one subject; (b) the site where blood was injected (ROI 1) and area where it was not (ROI 2) of one subject; (c) the RVI of ROI 1 and ROI 2 of one subject; (d) the RVI difference comparison before and after blood injection in statistical analysis of all subjects. (*∗∗ p* < 0.01); (e) the mean of WBRC from all subjects over time.

Figures 4(a), 4(b), and 4(c) demonstrate the changes in brain impedance on the EIT images when blood was injected (within 150 s)** in one subject with open cranium**. The impedance of the region where blood was injected gradually decreased as the injection proceeded.  Figure 4(b) defines the two ROIs: the site where blood was injected (ROI 1) and the area where it was not (ROI 2).  Figure 4(c) shows that the impedance of ROI 1 decreased with the amount of injected blood; the greatest decline lasted for the first 20 s. The impedance of ROI 2 slightly decreased as blood was injected. In statistical analysis (Figure 4(d)), the difference of impedance changes within ROI 1 before and after the blood injection was considered significant (*p* = 0.000042) but not within ROI 2 (*p*=0.1128); the impedance change of the entire course in ROI 1 (-0.033916) was approximately 10 times greater than that in ROI 2 (-0.0041). Moreover, Figure 4(e) exhibited that the whole brain impedance sharply declined within the first 20 s (the impedance changes can be up to 2.12±1.67%,* p*=0.000117); then, impedance gradually decreased as blood continued to be injected. (The overall change in impedance reached 4.06±1.95%,* p*=0.00007,)

### 3.4. EIT Imaging of the Blood Injection Procedure in Rabbits with Closed Cranium

**Figure 5:**
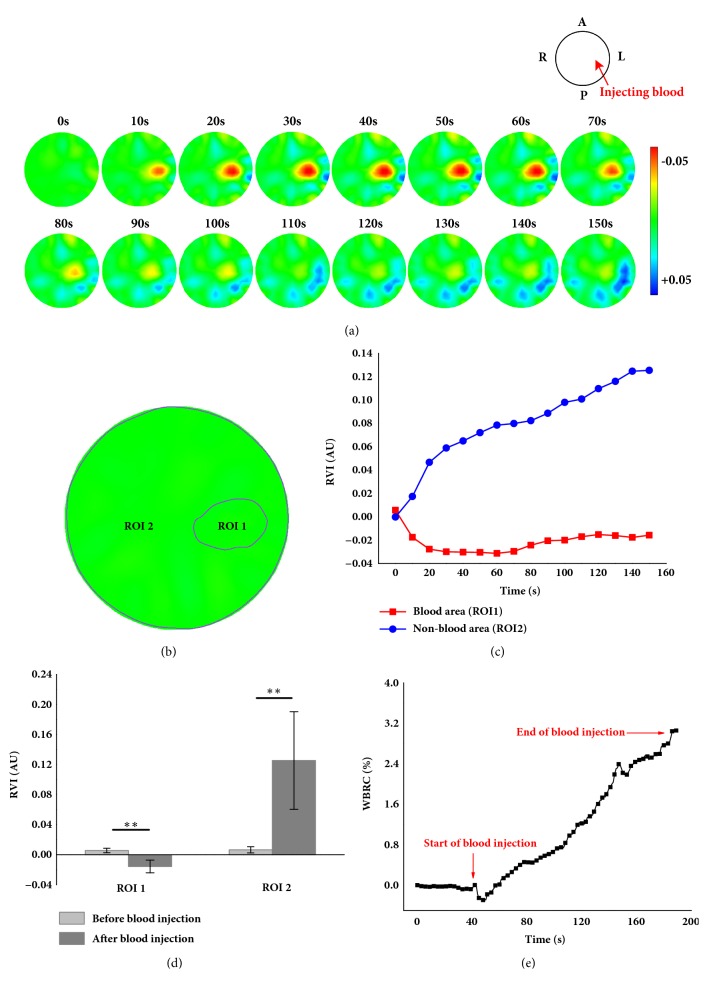
EIT imaging of the ICH procedure in rabbits with the closed cranium. (a) The reconstructed EIT images on one subject; (b) the site where blood was injected (ROI 1) and area where it was not (ROI 2) on one subject; (c) the RVI of ROI 1 and ROI 2 on one subject; (d) the RVI difference comparison before and after blood injection in statistical analysis from all subjects. (*∗∗ p* < 0.01); (e) the mean of WBRC from all subjects over time.

Figures 5(a), 5(b), and 5(c) depict the changes in brain impedance in the EIT images when blood was injected (within 150 s)** in one subject with closed cranium**. The impedance of the area where blood was injected decreased as the amount of injected blood increased within the first 70 s of starting the injection; the impedance of the area surrounding the site of the injection gradually increased.  Figure 5(b) also defines the ROI 1 where blood was injected and the ROI 2 where blood was not. As shown in Figure 5(c), the impedance of ROI 1 rapidly decreased within the first 20 s, remained almost unchanged for the next 30–60 s, and slightly increased between 70 and 90 s. The impedance of ROI 2 generally kept increasing as blood was injected, especially within the first 20 s. In statistical analysis (Figure 5(d)), it was noted that the differences of impedance changes in ROI 1 and in ROI 2 were both significant (*p* = 0.0046 for ROI 1 and* p* = 0.000073 for ROI 2) and the amplitude of impedance changes in ROI 2 (0.1254) was almost 6 times greater than that in ROI 1 (-0.0157). For the whole brain impedance, it fell by 0.3±0.21% at the start of blood injection (*p*=0.0025) and ultimately increased by 3.25±1.79% (*p*=0.00013) (Figure 5(e)).

## 4. Discussion

### 4.1. Summary and Explanation of Experimental Results

As we hypothesized that the cranium characteristics is one of the potential reasons leading to the different intracranial impedance changes when using EIT to monitor the ICH progression, this study investigated this effect of open or closed cranium on impedance changes within brain in the rabbit ICH model. Interestingly, a notable difference in intracranial impedance changes (including whole and regional impedance) was found between open and closed cranium conditions.

When the cranium was open, the regional brain impedance of injection site remarkably decreased as blood continued to be injected and the whole brain impedance also decreased by around 4%. This is largely in agreement with the results reported by Xu et al. [15] and Manwaring et al. [16]. However, Sadleir et al. [18] recorded an increase in brain impedance when a small amount of a blood-like fluid was injected into parenchyma near the left and right ventricles of a piglet cadaver. In their study, they used the decapitated piglet head when establishing the ICH model whereas we created the ICH model in rabbits* in vivo*. This fact may be the one reason for the different impedance changes within brain in that the* ex vivo* tissue would inherently lose its biological activity and thus its impedance varies over time [27].

When the cranium was closed, the impedance of blood injection site rapidly decreased at the beginning of injection and then remained almost unchanged; the impedance in the area where blood was not injected continued to increase. In addition, the whole brain impedance also slightly decreased at first and then remarkably increased at last. These results resemble the findings by Chen et al. [21] who induced a rabbit ICH model by injecting collagenase into white matter. Collagenase is a matrix metalloproteinase that breaks down and destroys collagen in the vascular basement membrane and cell matrix, disrupts the blood–brain barrier, and causes blood vessels to slowly bleed. They found a direct increase in brain impedance using EIT after injecting collagenase. Moreover, our results are in good accordance with the phenomenon observed by Yang et al. [22] who employed an impedance analyzer to measure the ICH progression in the closed cranium rabbit model and discovered that the whole brain impedance went down at first and then went up when injecting blood into cerebral parenchyma. However, Dowrick et al. [17] reported that brain impedance decreased after autologous blood was injected into the rat brain. The reasons for this difference remain unclear.

Intuitively, as blood impedance (**1.4 Ω·m**) is much smaller than the impedance of cerebral parenchyma (**8.5 Ω·m**) [14], it appears that the whole brain impedance should decline when blood is injected under both open and closed cranium conditions. Nevertheless, by comparing and analyzing the differences between open and closed cranium conditions, it is reasonable to speculate that the ICH model established under two different conditions might be physiologically distinct. The ICH model established when the cranium was open possibly maintains a constant intracranial pressure and perhaps the one when the cranium is closed maintains a constant intracranial volume, more similar to actual clinical ICH. When the cranium was open and the blood was injected into the cerebral parenchyma, the brain tissues at the injection site were displaced by blood and moved to the surrounding area due to higher pressure. Since the cranium was no longer intact, it did not restrict the movement of brain tissues. To keep a constant pressure, the brain tissues at the injection site would always move to an area of lower pressure. Therefore, both whole brain impedance and regional blood impedance at the injection site would decrease. When the cranium was closed, the injected blood pushed the surrounding brain tissue away due to the higher pressure at the injection site. Apparently, blood occupied the area of the brain tissue and whole brain impedance should also decline. However, because the cranium is intact in the closed cranium ICH model, the cranial compartment is incompressible and the volume inside it is fixed; the cranium, blood, cerebrospinal fluid (CSF), and brain tissue create a state of volume equilibrium. Therefore, any increase in volume of one of these cranial components must be compensated by a decrease in volume of another (Monro–Kellie's hypothesis) [28]. This fixed volume only allows brain tissues to move within the cranial cavity. When the blood is injected, it pushes the brain tissues to the area with the lower pressure while the brain tissues may squeeze and displace the surrounding CSF at the same time. Therefore, we presume that, in this study, the impedance of the area where blood was injected decreased because blood impedance was lower than the impedance of parenchyma whereas the impedance of the area surrounding the blood injection site and the whole brain impedance increased because the impedance of parenchyma is greater than that of CSF (0.8 S/m).

### 4.2. Implication for ICH Monitoring with EIT

Given our results in this study, the condition of the cranium is indeed a key factor when using EIT to monitor ICH. The results of using EIT to monitor ICH when the cranium was open or closed were clearly dissimilar. Hence, when conducting further researches into using EIT to monitor ICH, we suggest that intactness of the cranium, especially in animal experiments, be guaranteed as much as possible in attempt to simulate ICH occurring in patients in clinical settings because the cranium of most ICH patients is complete except for those undergoing craniotomy. Plus, as both the impedance at the site of bleeding and at the area around it changed, it is also suggested that in future studies EIT images be divided into different areas to analyze global and regional impedance changes caused by ICH.

As far as the biological impedance properties concerned, blood might be another potential factor. Lei et al. [29] showed that if autologous blood was not heparinized and did not start to coagulate, its impedance is close to heparinized blood (approximately 0.71 S/m); once the autologous nonheparinized blood began to clot at around 1500s* ex vivo* (at 37°C), its impedance dramatically increased. In the published literatures about the use of EIT to monitor ICH, all ICH models were established within 1500s, so we presume that the differences in the previous results may not have been caused by the impedance properties of blood, no matter blood is heparinized or not. Certainly, this hypothesis needs further confirmation in future study. However, if EIT is going to be used to monitor ICH in the long term in the clinical setting, then the characteristics of blood must be taken into account.

Besides the cranium and blood, the injection site may also result in the different impedance changes within brain. For instance, when the blood was injected into cerebral ventricle [19] or subarachnoid space [20], which is full of CSF, the corresponding impedance gain was observed on EIT images; the possible reason may lie in the lower impedance of CSF than blood. Thus in future study, it is necessary to be aware of the blood injection site and consider the interaction among blood, parenchyma, and CSF, as well as their effects on the relevant impedance changes of brain.

### 4.3. Study Limitations

We acknowledge that our study has certain limitations. When establishing the ICH models, the changes in the intracranial pressure of each animal were inferred. However, without a direct measurement of the intracranial pressure, such an assumption cannot be validated. In future, intracranial pressure should be measured to further analyze the relationship between injected blood, intracranial pressure, and EIT images. Moreover, this study focused on whether the cranium condition had influences on the impedance changes as blood was being injected into the cerebral parenchyma. In the clinical practices, ICH often needs to be monitored in the longer run, and it is thus necessary to investigate the impedance changes on EIT images caused by ICH from the start of bleeding to several hours after bleeding. In future, the duration time of using EIT to monitor ICH should be increased to further study subsequent changes in impedance on EIT images.

## 5. Conclusion

In order to investigate the effects of the cranium completeness (open or closed) on the impedance changes within brain in the ICH model, we employed EIT to continuously observe two ICH models (open or closed cranium) in rabbits. Results showed that when the cranium was open, the impedance of the area where the blood was injected, as well as the whole brain impedance, decreased with the amount of blood being injected; when the cranium was closed, while the impedance of the area where blood was not injected continued to increase, the impedance of the area where blood was injected decreased within 20 s of the blood being injected and then remained almost unchanged, and the whole brain impedance had a small fall and then notably increased. This study further validates the feasibility of monitoring ICH with EIT and provides helpful suggestions for future researches on application of EIT to monitor ICH, including establishing an ICH model under closed cranium condition, considering the impedance properties of blood in long-term monitoring and the exact intracranial site of injecting blood.

## Data Availability

The EIT data used to support the findings of this study are included within the article.
